# Antioxidant, Antimicrobial and Metmyoglobin Reducing Activity of Artichoke (*Cynara scolymus*) Powder Extract-Added Minced Meat during Frozen Storage

**DOI:** 10.3390/molecules26185494

**Published:** 2021-09-09

**Authors:** Tuğba Demir, Sema Ağaoğlu

**Affiliations:** Department of Food Hygiene and Technology, Faculty of Veterinary Medicine, Sivas Cumhuriyet University, Sivas 58140, Turkey; sagaoglu@cumhuriyet.edu.tr

**Keywords:** meat quality, antioxidant activity, antimicrobial activity, functional food, *C. scolymus*, food quality

## Abstract

The present study aimed to investigate the bioactive compounds in artichoke (*Cynara scolymus*) powder, having antioxidant and antimicrobial activity, and to determine the effectiveness of artichoke (*C. scolymus*) powder extract within the minced meat. *C. scolymus* was extracted using two different methods. The method incorporating high phenolic and flavonoid content levels was used in other analyses and the phenolic and flavonoid contents in *C. scolymus* extract was determined using LC-QTOF-MS. Antioxidant, antimicrobial, and metmyoglobin (metMb) reducing activities and pH values of the extract-added minced meat samples were measured for 10 days during storage. DPPH, FRAP, and ABTS were used in the antioxidant analyses. The antimicrobial activity of *C. scolymus* extract was evaluated on five different food pathogens by using the disc diffusion method. The most resistant bacterium was found to be *Listeria monocytogenes* (18.05 mm ± 0.24). The amount of metMb was measured in the minced meat sample that was added to the extract during storage (*p* < 0.05). MetMb formation and pH value on the sixth day of storage were found to be at lower levels than in the control group. In conclusion, *C. scolymus* exhibited a good antimicrobial and antioxidant effect and can be used in storing and packaging the food products, especially the meat and meat products.

## 1. Introduction

Meat is one of the foods with the highest protein content and provides essential amino acids needed in human nutrition. Biochemically, meat contains proteins, essential amino acids, water, and low amounts of minerals, vitamins and carbohydrates [1]. The oxidative degradation of lipids during processing and storage affects the quality characteristics of meat and meat products. Primary and secondary oxidative decomposition products reduce the nutritional quality of meat, and they create an important health risk [2].

As a result of the advancing technology and increasing fast-food consumption, natural or synthetic antioxidants are used in order to expand the shelf life and enhance the quality properties of meat and meat products.

Antioxidants are defined as the compounds that are capable of binding the free radicals to give hydrogen (H·) radicals in order to prevent the oxidation reaction [3]. 

Antioxidative agents delay the oxidative degradation of lipids, increase the quality, and maintain the nutritional value. These substances prevent cell damage and tumor formation by neutralizing free radicals [4]. In recent years, synthetic antioxidants in meat and meat products were aimed at increasing the preservation time and improving the quality [3]. However, there also are safety concerns about the use of synthetic antioxidants. Consumers consider the natural antioxidants more acceptable than the synthetic ones. Natural antioxidant agents delay the lipid oxidation reaction and the quality and shelf life can preserve without damaging the nutritional value of the meat [5].

Meat and meat products offer a suitable environment for the growth and propagation of bacterial spoilage and foodborne pathogens. Meat and meat products can be contaminated by pathogenic bacteria in any of the steps between the “farm and the fork”, such as processing, packaging, and pre-cooking storages [6]. For this reason, hygiene and preservation methods are reinforced using natural bioactive compounds in the meat industry [7].

The antimicrobial action mechanisms of plant components differ from those of synthetic antimicrobials, and they inhibit the bacterial growth through a series of metabolic reactions [7,8]. Phenolic compounds commonly found in their structures show an antimicrobial effect by lowering the intracellular pH, by chelating some metals that are necessary for the survival of microorganisms, or by changing the permeability of the cell membrane and disrupting the substrate transport [9,10]. Plant-based extracts protect meat and meat products against bacteria, which cause spoilage and expand preservation time and nutritional value thanks to their phenolic and flavonoid compound contents. Natural phenolic compounds also play an effective role in taste and flavor, since they have at least one aromatic ring [11]. Previous research showed that the addition of antioxidants to fresh red meat inhibited lipid oxidation and delayed metMb formation. They reported that the antioxidants preserved the fresh meat color by preventing the lipid oxidation of the hemoproteins and/or acting on the enzymic reducing systems [12].

Artichoke (*Cynara scolymus*) is a plant that is widely grown and consumed throughout the world. *C. scolymus* has a strong potential in terms of scavenging the reactive oxygen species and free radicals, which contain many natural compounds. In many studies, it was emphasized that the caffeoylquinic acid derivatives and luteolin and apigenin glycosides found in the structure of *C. scolymus* showed strong antioxidant effects [13]. There also are many studies underlining the antibacterial and antioxidant properties of *C. scolymus* extract. The bioactive property of artichoke is related to its high-level luteolin and chlorogenic acid contents [14]. The main phenolics of *C. scolymus* are cinnamic acid derivatives including caffeic acid, cynarin, 1,5-*O*-di-o-cafeoylquinic acid and 3,4-*O*-di-o-cafeoylquinic acid and 1,5-*O*-di-o-cafeoylquinic acid, whereas the major flavonoids were reported to be 4′,5,7-trihydroxyflavone (apigenin), 3′,4′,5,7-tetrahydroxyflavone (luteolin), and glycosidic derivatives [15]. There are limited studies on marinating meat and meat products with *C. scolymus*. The present study aims to extract and characterize the total phenolic and total flavonoid compounds from *C. scolymus* powder applied to minced meat in order to improve quality characteristics during the storage process. For this purpose, antioxidant, antimicrobial, and metMb reducing activities of artichoke powder extract added into minced meat were investigated.

## 2. Materials and Methods

### 2.1. Materials

Artichoke flowers (*C. scolymus*) were obtained from an organic market, ground and stored at +4 °C until analyzed. Minced beef was obtained from a local butcher in Sivas, Turkey. Mueller–Hinton Broth (MHB) and Mueller–Hinton Agar (MHA) were obtained from Merck (Merck KGaA, Darmstadt, Germany). The microorganisms (*Enterococcus faecalis* (ATCC 029212), *Escherichia coli* (ATCC 025922), *Staphylococcus aureus* (ATCC 029213), *Listeria monocytogenes* (ATCC 07644) and *Salmonella typhimurium* (ATCC 014028)) were provided from the Microbiology Laboratory of Sivas Cumhuriyet University Research Hospital. The ampicillin disc was obtained from Oxoid (Oxoid Ltd., Thermo Fisher Scientific, Inc., Basingstoke, Hampshire, United Kingdom). The phenolic compounds were purchased from Sigma-Aldrich (St. Louis, MO, USA). All other chemicals used are of analytical standard and obtained from Merck (MerckKGaA, Darmstadt, Germany) or Sigma-Aldrich (USA).

### 2.2. Preparation of the C. scolymus Extract

We used mature artichoke flowers (*C. scolymus*) in an organic market from the Ege Region of Turkey. The drying process was carried out in a lyophilizer for 24 h in the following conditions: pressure 0.945 mbar, initial temperature −30 °C, final temperature +30 °C. After drying, the samples were ground in a laboratory mill to a fine powder and stored vacuum-packed in a freezer at −80 °C until analysis. Powdered samples (ratio; 1:3 g/mL water) were extracted for 24 h in the Soxhlet extractor (30 g plant: 90 mL distilled water). This group was filtered and used directly (Method 1). In another group, the extract was concentrated by a rotary evaporator under a low pressure (174 mbar) and controlled temperature (40–50 °C) for 4 h (Method 2). The working condition with the highest phenolic and flavonoid content (Method 2) was chosen for chromatographic analysis. All analysis were performed in triplicate. All methods were performed in accordance with the relevant guidelines and regulations (institutional, national, and international guidelines and legislation for use of plant material). 

### 2.3. Determination of Total Phenolic and Flavonoid in the Extract

The total phenolic content of the extract was determined according to the method described using the Folin–Ciocalteu phenol reagent [16]. The quantitative determination of total phenolic content of extract was obtained by mixing 100 μL of 2 N Folin–Ciocalteu’s phenol reagent with 100 μL of extract/100 μL of the gallic acid solution 1 mL of 7% sodium carbonate, and 2.3 mL of water. A standard curve was created with gallic acid (0−0.5 mg/mL). Then, (25 °C, 2 h), the absorbance at 750 nm was measured in the spectrophotometer. The determination of the total amount of the flavonoid of the extracts were applied according to the method provided by Zhishen et al. [17]. The extract was reacted with in order of 5% NaNO_2_, 10% AlCl_3_ and NaOH (1 M). Quercetin was used as a standard to determine the total flavonoid content of the extracts. A standard curve was created with quercetin (0−100 mg/L) and the absorbance was measured at 510 nm. Total phenolic compounds were expressed as gallic acid equivalent (GAE), and total flavonoid compounds as quercetin equivalent (QE). All analysis were performed in triplicate.

### 2.4. Characterization of Phytochemical Composition with LC/QTOF-MS

The phenolic and flavonoid contents in extract of *C. scolymus* was characterized with LC-QTOF-MS. Phytochemical composition was defined using an Agilent Technologies 6530 OHD Accurate-Mass Q-TOF-MS&MS system (Agilent Technologies, Santa Clara, CA, USA) accoutered with the Agilent 1290UPLC system and an electrospray ionization source. For the characterization of phytochemical compounds, many parameters were analyzed and applied in combination (ion, molecular weight, fragmentation pattern, retention time, LOD and LOQ). Method calibration and validation parameters for analysis of the phenolic standards are presented in Table 2 and compared with reference data. Characterizations of components were made using formic acid (0.1%) and acetonitrile (0.1%) as mobile phase. The flow rate was 0.5 mL/min while the sample injection volume was 0.5 μL. Analysis were made on the programmed MS/MS system. The collision energy conditions are given below. Conditions; *m*/*z* 0−200, 20 eV, (0−10); *m*/*z* ≥ 200, 3 eV, (10−20); *m*/*z* ≥ 400, 40 eV, (20−30); *m*/*z* 600−700, 50 eV, (30−40); *m*/*z* ≥ 700, 60 eV, (40−50). Finally, MS data were operated with software. MS spectra of the compounds were identified by comparison with standards [18].

### 2.5. Preparation of Meat Samples

Beef rounds (*Semimembranosus* muscle) from different steers (*n* = 4) were purchased from a local butcher (Sivas, Turkey) at 3 days postmortem. The lean beef round was ground using a meat grinder through a plate with an 8 mm steel plate twice. After mixing, the minced meat round was divided into 2 batches (approximately 1 kg/batch) for study. The minced meat without fat was divided into two groups which are for control and treatment. Twenty-four samples (two groups) of 30 g of raw minced meat were placed in plastic bags. Twenty samples of 30 g of minced meat were added with the extract. Other samples (4 samples) were used as negative controls (not treated with *C. scolymus*). Minced meat in the treatment groups were immersed in 8% (*v*/*w*) of extract [19]. Treatment and control groups were stored (−18 °C) before being ready to be analyzed for a storage period (day 0, day 3, day 6, day 10). Each test was performed in triplicate.

### 2.6. Antimicrobial Activity

Antimicrobial activity of *C. scolymus* extract was defined by the agar diffusion method [20]. *E. coli, S. aureus, L. monocytogenes, S. typhimurium and E. faecalis* pathogens used. The fresh inoculum of bacteria (10^5^ cfu/mL) was prepared in sterile-saline water (2 mL). The turbidity of inoculum suspensions were set to a 0.5 McFarland standard. Sterile antimicrobial discs (6 mm) were impregnated with 30 μL of *C. scolymus* extract, followed by waiting until the disk absorbed the extract. Distilled water was used as the negative control, and ampicillin as the positive control. Finally, all treatment plates were left for 10 min at 25 °C to allow the diffusion of the plant extract, and all plates were last-incubated at 37 °C for 24 h. The diameters of the inhibition zones were measured after the incubation period. Measurements were conducted in triplicate.

### 2.7. Preparation of Minced Meat Agar-Solution

Peptone water (90 mL) was added (buffered) to 10 g of minced meat. The prepared mix was vortexed and blended for 5 min. It was blended until no particles were left and smoothed. After that, the solution was centrifuged (5000× *g*, 10 min) to obtain the clarified extract by removed the solid particle. The final solid media with agar (1.5%) was prepared until 1 L. Then, prepared meat agar solution were autoclaved (121 °C, 15 min) [18,21].

### 2.8. Antioxidant Activity

#### 2.8.1. DPPH: 2,2 Diphenyl-1-picrylhydrazyl Radical Scavenging Activity

Free radical scavenging activity was evaluated by the DPPH assay using the standard method [22]. Minced meat with added extract (0.05 g) was continually mixed with 3 mL of DPPH working solution (1.95 mL, 100 µM) that has been prepared using methanol in a test tube at 25 °C for 10 min. Then, all treatments were centrifuged (1500× *g*, 10 min). Trolox solution was used as standard. By using methanol as blank, absorbance of the supernatants was measured at 517 nm. All results were expressed as µmol “Trolox Equivalent” (TE) after constructing a TE standard curve. Measurements were performed in triplicate.

#### 2.8.2. TEAC Trolox Equivalent Antioxidant Capacity

Antioxidant activity of the samples was also measured using an improved ABTS procedure [18]. The ABTS radical cation (ABTS+) solution (7 mM ABTS, 2.45 mM) was prepared through the reaction potassium persulphate, then the pre-incubation in the dark for 20 h at 25 °C. The ABTS+ solution was then diluted with 80% ethanol to obtain an absorbance of 0.700 ± 0.005 at 734 nm. Then, 2.9 mL of ABTS working solution at absorbance 700 nm was added to 0.05 g of the treatments and the solution was mixed strongly. This working mixture was stored at 30 °C for 25 min, then centrifugated at 1500× *g* for 10 min. Trolox solution was used as standard. The supernatant’s absorbance was measured at 734 nm. All results were expressed as µmol “Trolox Equivalent” (TE) after constructing a TE standard curve. Measurements were performed in triplicate.

#### 2.8.3. FRAP: Ferric Reducing Antioxidant Power

Minced meat with 0.05 g was mixed with 2.5 mL of 200 mM sodium phosphate buffer (pH 6.6) and 2.5 mL of 1% potassium ferric cyanide {K_4_[Fe (CN)_6_]} and incubated at 45 °C for 30 min. Then, 2.5 mL of 10% trichloroacetic acid (*w*/*v*) was added on mix. The mixture was centrifuged at 1500× *g* for 10 min and deionized water was added with an equal volume of resulting supernatant and 1/5 volume of 0.1% FeCl_3_ and stored at 25 °C for 10 min. Absorbance of mix-solution was read at 700 nm [21]. Measurements were performed in triplicate.

### 2.9. Measurement of metMb Reducing Activity

Firstly, 0.5 g minced meat was homogenized with added 3 mL phosphate buffer (0.04 M; pH 6.8), and was stored at a cooled temperature (4 °C). The homogenate was centrifuged at 2000× *g* for 10 min and left to pre-incubate at 4 °C for 1 h. MetMb accumulation in minced meat was evaluated by spectrophotometrically as defined by Huang et al. [19]. The homogenate was centrifuged at 2500× *g* for 5 min at 4 °C and the supernatant filtered through Whatman No. 1 filter paper to remove fat. The standard mixture contained 0.1 mL EDTA (5 Mm), 0.1 mL phosphate buffer (50 mM; pH 7.0), 0.1 mL [K_4_ Fe(CN)_6_]; 3.0 mM, 0.1 mL water, and 0.2 mL 0.75 metMb Fe(III) in 2.0 mM phosphate buffer (pH 7.0). MetMb reducing activity was calculated from a calibration curve using standard mixture. Finally, metMb reducing activity was estimated from the absorbance at 580 nm. Measurements were performed in triplicate.

### 2.10. Instrumental Color Measurements

Instrumental color analysis was executed using a Hunterlab colorimeter (Mini Scan XEPlus, Virginia, WV, USA). Before each application, the colorimeter was calibrated on the CIE (Commission internationale de l′éclairage) color space scheme using a tile (black and white). The L* value designates lightness (L* = 0−100; darkness–lightness); a* value designates redness ((+) 60; red, (−) 60; green)) and b* value designates yellowness ((+) 60; yellow, (−) 60; blue). Color measurements were taken at 4 °C with illuminant D65 and a 0° angle observer. All measurements were taken on the outer surface of minced meat from randomly chosen selected locations [23]. Measurements were performed in triplicate.

### 2.11. pH Determination

For the pH value measurement, a 1.00 g sample was homogenized by adding 5 mL sterile-distilled water and the homogenate was centrifuged at 2000× *g* for 10 min. The supernatant was filtered through Whatman No. 1 filter paper, and the pH of the supernatant recorded using a pH meter (Hanna Edge, Hanna Instruments, Woonsocket, RI, USA).

### 2.12. Statistical Analysis

All the tested sample data (mean values) were statistically analyzed with the SPSS analysis of variance (SPSS version 19.0 software, SPSS; Chicago, IL, USA). A Duncan’s multiple range test was used for the study of studentized range distribution in order to determine critical values for comparisons between means. The significance for all comparisons were determined at the *p* < 0.05 level.

## 3. Results and Discussion

### 3.1. Total Phenolic and Flavonoid Analysis in Extracts

Phenolic compounds are considered to be natural sources of antioxidants required by metabolism, as well as their antioxidant activities; they are demonstrate by binding free radicals or chelating with metals [2]. These effects increase with the increase in the number of OH groups in the phenol ring they contain in their structure. Moreover, phenolic compounds have the ability to delay, slow, or prevent the oxidation at low concentrations and to remain in a stable form when converted to free radicals [11]. In the present study, the phenolic compounds were calculated using standard gallic acid, and total flavonoid content was measured using standard quercetin. Quercetin and derivatives are available in various foods and plant-based products. Quercetin and gallic acid were reported to play effective roles against oxidative stress. Quercetin is among the compounds that play a role in the inhibition of lipoxygenase that is responsible for inflammation [9].

Total phenolic and total flavonoid contents of *C. scolymus* powder extracts, which were extracted using one of the extraction methods, are presented in Table 1. Calculations were made on the standard curve formed by the measured absorbance values (y = 1.8902x + 0.0128, R^2^ = 0.99; y = 0.0039x − 0.003, R^2^ = 0.99). The results obtained using evaporator were higher in terms of both phenolic and flavonoid contents. In the literature, the amount of bioactive components passing into the solvent in concentrated extracts was reported to be high [10].

A past study about total phenolic content in ethanol extracts of the artichoke demonstrated that the extracts were rich in total phenolic (32.2 mg GAE/g) [24,25]. Curadi et al. [26] found that total phenolic content in artichoke byproducts were found to be between 7.31 and 13.05 mg/g in the methanol extracts as chlorogenic acid equivalent. In this other study, the highest phenolic content of artichoke extract was determined as 4.39 mg GAE/100 g extract [23]. In another study, total phenolics were found to be between 1.60−9.80% of chlorogenic acid, equivalent in different species of artichoke [27]. When all the findings are interpreted together with the results of this study, our study are parallel to the literature studies.

Gallic acid is a phenolic acid and it belongs to the hydroxyl-benzoic acid group. It has a high level of antioxidant and antimicrobial activity. Gallic acid is a natural antioxidant substance that can be isolated from plants and is used in a wide range of medications and cosmetic products, as well as usage in the food industry [9]. Gallic acid can be used as a food additive, especially in food preservation, in food technology. The bioactive role of this phenolic acid is to prevent rancidity in fats and oils. Many studies examine natural food additives [4]. Comparing the antioxidant capacities of natural and synthetic antioxidants, gallic acid showed properties close to natural antioxidants (BHT, BHA) [6]. For this reason, gallic acid and quercetin were used as standards in determining total phenolic and total flavonoid compounds, and the results were calculated as gallic acid and quercetin equivalents. As can be seen in Table 1, the extraction method affects the amounts of total phenolic and total flavonoid substances. Extraction method, method validation, and extraction under optimum conditions are among the purposes of obtaining the highest amount of phenolic compounds. In the present study, the extract obtained using a rotary evaporator was used in all the experiments below.

### 3.2. Identification and Quantification of Polyphenols by LC-QTOF-MS

**Figure 1:**
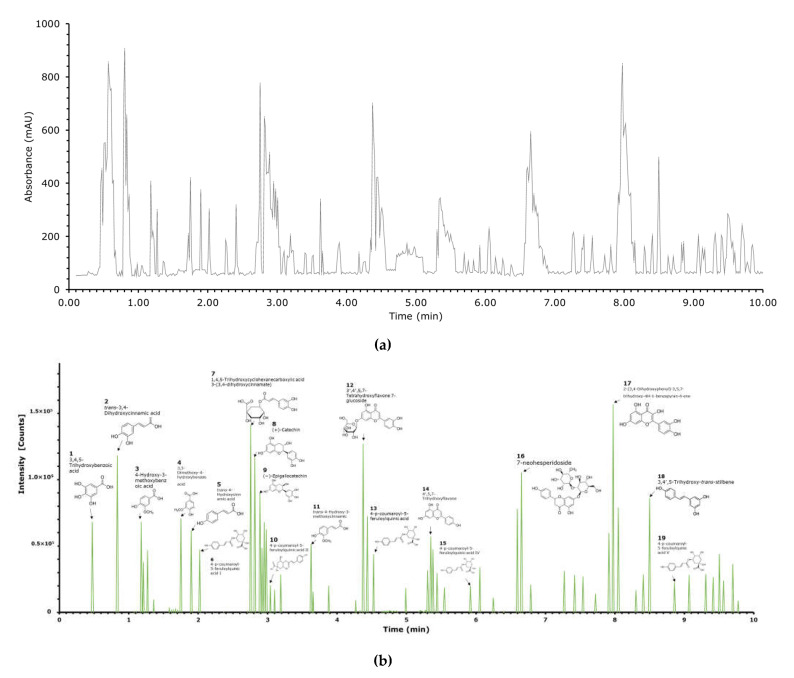
LC-QTOF-MS (**a**) photodiode array detector analysis spectra (**b**) MS spectra analysis of *C. scolymus* powder extracts.

**Figure 2:**
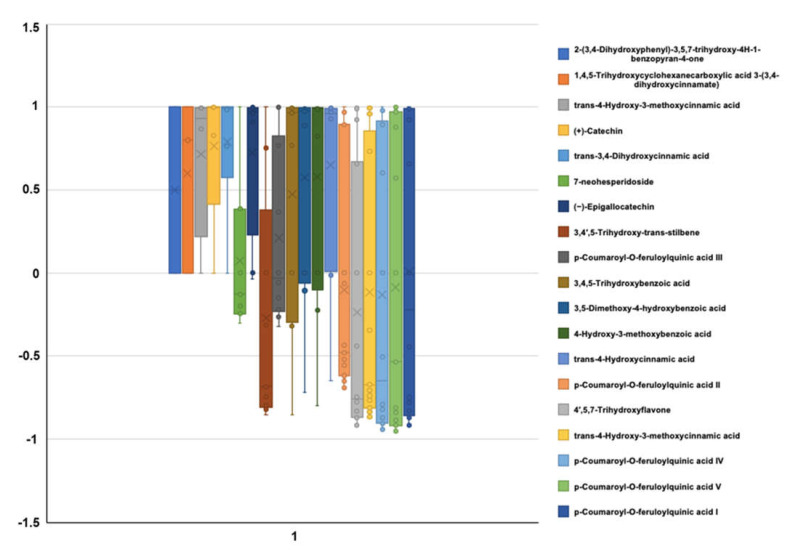
Correlation matrix of the obtained phenolic compound compositions.

LC-QTOF-MS analysis was performed to specifically identify and characterize phenolic and flavonoid compounds found in *C. scolymus* extracts. In addition, the bioactive components of the extract were determined quantitatively and using standards (Figure 1). Figure 1 shows LC-QTOF-MS photodiode array detector analysis spectra (Figure 1a) and MS spectra analysis of *C. scolymus* powder extracts (Figure 1b). Table 2 shows method calibration and validation parameters for analysis of the phenolic standards, and Figure 2 illustrates the correlation matrix of the phenolic compound compositions. The linearity range as well as the slope and the intercept of calibration graph with the respective square of correlation coefficient (R^2^) was defined for each of the 19 phenolic compounds (Table 2). The R^2^ value of each phenolic compound analyzed was found to be higher than 0.9964, and this result indicates the high correlation of data in the concentration range analyzed here. The use of higher concentrations for the chosen phenolic compounds significantly decreased their linearity. The correlation matrix of the phenolic compound composition obtained is presented in Figure 2. Pearson’s correlation analysis was performed to determine the direction and level of the relationship between phenolic compounds. A correlation coefficient between the variables close to −1 indicates a strong negative relationship, whereas those closer to +1 indicate a strong positive relationship. Values closer to zero indicate that there is no significant relationship.

In recent years, LC-QTOF-MS chromatography can be used in many fields, especially for identifying the bioactive compounds [27]. When the analysis results were evaluated, a total of nineteen components were identified and their chemical formulations were presented (Figure 1). Quercetin [2-(3,4-Dihydroxyphenyl)-3,5,7-trihydroxy-4H-1-benzopyran-4-one] was found at the highest density (11.63%). In addition, the following compounds were identified, respectively; chlorogenic acid [(1,4,5-Trihydroxycyclohexanecarboxylic acid 3-(3,4-dihydroxycinnamate)] (11.18%), luteolin (trans-4-Hydroxy-3-methoxycinnamic acid) (10.49%), (+)-catechin (8.75%) and caffeic acid (7.79%) (trans-3, 4-dihydroxycinnamic acid). With the addition of quercetin to meat products, the antioxidant potential has increased and lipid peroxidation and fatty acid composition were improved [28]. On the other hand, quercetin had the potential of improving the quality of meat products. This process improved storage stability and inhibited the formation of lipid oxidation products, which was associated with quercetin [29]. Another bioactive compound having a positive effect on meat quality is chlorogenic acid. This compound improves meat quality and oxidative stress [30] and can be used as an antioxidant and antimicrobial agent in meat products [9]. Besides the antioxidant properties of luteolin (trans-4-Hydroxy-3-methoxycinnamic acid), catechin and caffeic acid, which were isolated from plants, these compounds also have anti-inflammatory and anticancer activities [15]. The other compounds identified and quantified in the present study are as follows; 7-neohesperidoside (7.05%), (−)-Epigallocatechin (5.95%), 3,4′,5-Trihydroxy-trans-stilbene (5.70%), 3,4,5-Trihydroxybenzoic acid (4.0%), 3,5-Dimethoxy-4-hydroxybenzoic acid (3.86%), 4-Hydroxy-3-methoxybenzoic acid (3.72%), trans-4-Hydroxycinnamic acid (3.62%), 4′,5,7-Trihydroxyflavone (2.50%), trans-4-Hydroxy-3-methoxycinnamic acid (2.30%), p-Coumaroyl-*O*-feruloylquinic acid I-V (11.45%).

### 3.3. Antimicrobial Activity

The antimicrobial activity of *C. scolymus* extract was examined on five different food pathogens. In selecting the pathogens, the risk on meat and meat products was determined among the microorganisms. All the analyses were performed using the agar disk diffusion method. Two concentrations of the extract were used to measure the antimicrobial activity of the minced meat (5% and 10%). Evaluating the results, the most resistant bacterium was found to be *L. monocytogenes* (18.05 ± 0.45) (Table 3). The extract showed the highest inhibition on *S. typhimurium* (30.95 ± 0.47), followed by *E. faecalis* (25.37 ± 0.53), *S. aureus* (21.10 ± 0.92) and *E. coli* (20.07 ± 0.66). As the concentration of extract increased (*C. scolymus* (10%)), the zone of inhibition increased. Standard ampicillin disc was used as positive control and distilled was used as negative control. Results are presented in Table 3.

There are many studies reporting that the five pathogen bacteria selected in our study cause food poisoning and foodborne infections and intoxications. Various inhibition rates were achieved in pathogens [5,6]. Stop the development of microorganisms and prevent a secondary infection are the expected properties of plant-based antimicrobial agents [31]. The antimicrobial activity of extracts can be attributed to the identified phenolic compositions. A high level of correlation was found between antimicrobial properties and phenolic and flavonoid compounds (r = 0.9678; *p* ≤ 0.001). Meat and meat products are exposed to many potential risks in the post-slaughter production process. These risks include foodborne pathogenic microorganisms and environmental equipment contamination [29,32]. The meat products added with polyphenols such as luteolin-7-*O*-rutinoside, caffeic acid, quercetin and epigallocatechin-3-gallate can reduce these complications [32]. They are responsible for microbial inactivation depending on the position of the hydroxyl groups of phenolic compounds. Therefore, the more phenolic compounds the extract contains, the higher the effect will be. Thus, the amount of phenolic compound in extract plays an effective role in scavenging the food pathogens [13]. Nineteen different phenolic compounds were identified in the present study. The high-level antimicrobial activity is associated with this finding.

### 3.4. Antioxidant Activity

**Figure 3:**
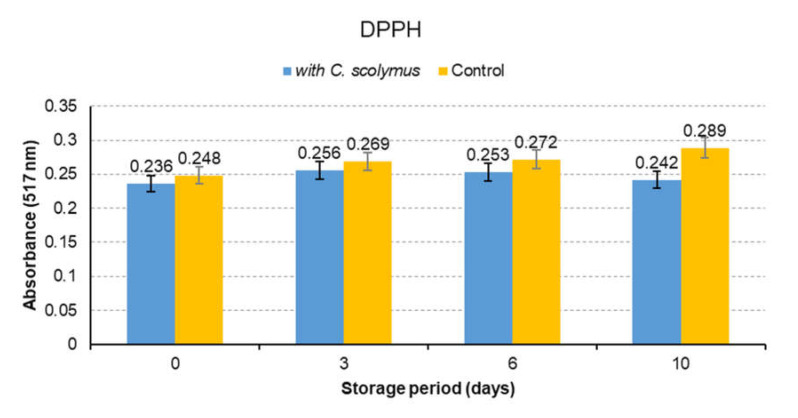
The antioxidant activity of control and marinated meat samples (DPPH assay).

**Figure 4:**
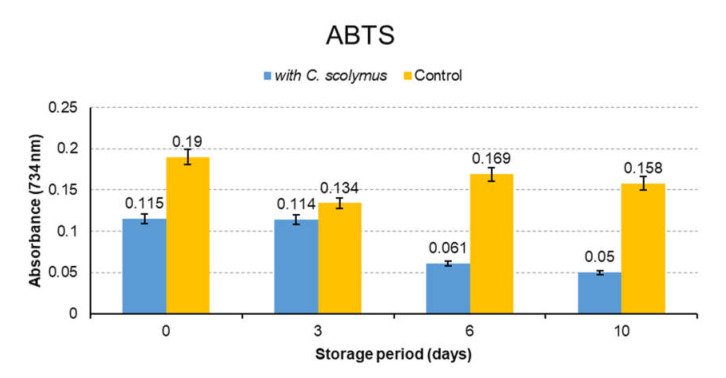
The antioxidant activity of control and marinated meat samples (ABTS assay).

**Figure 5:**
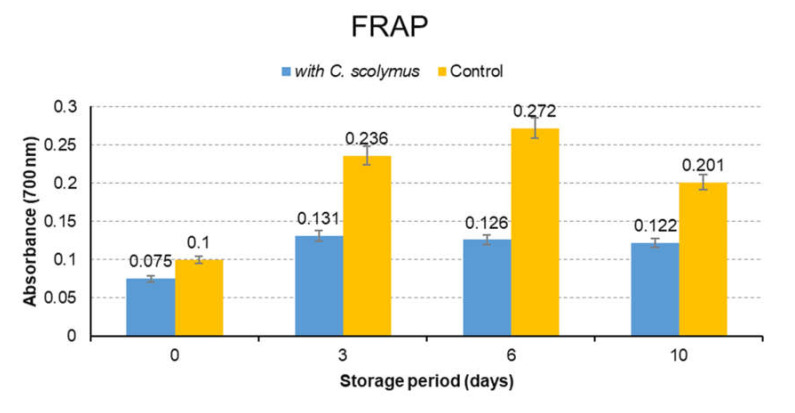
The antioxidant activity of control and marinated meat samples (FRAP assay).

Antioxidant activity of *C. scolymus* extracts was determined by DPPH, ABTS and FRAP methods. The analysis were completed in 10 days and run in triplicate. Absorbance readings were made on days 0, 3, 6 and 10 and average values were calculated. The results are presented in Figure 3. As seen in Figure 3, the samples containing *C. scolymus* showed a statistically significant difference since the third day comparison to the control group (*p* < 0.05). The decrease in the value of absorbance indicated the increase in antioxidant activities (Figure 3 and Figure 4). ABTS results were found to be higher than DPPH results (Figure 4). This finding might be explained by the faster electron transfer of ABTS• radical reactions when compared to DPPH. Figure 5 shows that increased absorbance of the reaction mix-solution increased the reducing power and antioxidant capacity (*p* < 0.05). Another useful and fast screening method for measuring antioxidant capacity is the FRAP method. The phenolic compounds found in *C. scolymus* extract are capable of blocking the radical chain reaction during the oxidation process and converting the free radicals into stable molecules by donating free electrons or hydrogen [13]. Various spectrometric and chromatographic studies have confirmed that quercetin and catechin are effective scavengers of singlet oxygen and peroxyl radicals [28]. The studies about *C. scolymus* extracts have reported their strong radical scavenging activities [23,33]. In the present study, the high antioxidant capacity of extracts is thought to be because of high quercetin and catechin content. A past study reported high antioxidant activity toward DPPH (8.3–49.7%), which was correlated with their polyphenol contents (1.7−9.86%) in the different artichoke samples [15]. Moreover, the other study about the artichoke byproducts showed a relatively high free radical scavenging activity and capacity to inhibit lipid peroxidation in artichoke byproducts [34]. The differences in antioxidant activities between this study and previous studies can arise from the use of different antioxidant measurement methods, different species (family) and different extraction conditions.

### 3.5. Measurement of metMb and Color Measurements

**Figure 6:**
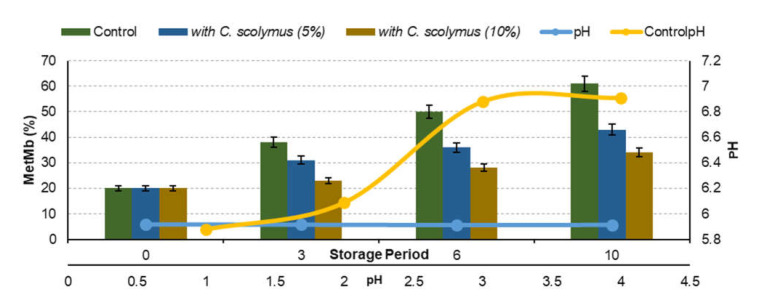
MetMb (%) and pH changes in meat samples marinated with *C. scolymus* powder.

The amount of metMb is correlated with the level of protein oxidation in the meat products. The amount of metMb increases with the storage time [18]. Two extract concentrations were used in measuring the metMb reducing activity of the minced meat (5% and 10%). Figure 6 illustrates the metMb values. As can be seen in Figure 6, the highest metMb ratios were observed in extract-added samples. Amounts of extract-added samples during storage are different compared to the control group. The visual sign of freshness and quality is the red color of meat. The reduction of red color with the formation of brown metMb occurs in parallel with the oxidative degradation of meat during storage [12]. MetMb is a pigment that is undesirable to occur on the meat surface. When an amount (60%) of the myoglobin present in meat is converted to metMb, the brown color of the meat can be detected by the eye [27]. Discoloration of meat occurs during the oxidation process because the aftereffect of lipid oxidation is the formation of pro-oxidants capable of reacting with oxymyoglobin, which leads to the formation of metMb [35]. Long-term storage at refrigeration temperatures or short-term storage at high temperatures causes surface drying; salt concentration increases and metMb formation is encouraged [18]. Liu et al. [36] reported that, compared to the control group, the treatment of beef patties with added natural antioxidants (vitamin E, carnosine, grape seed extract and tea catechins) resulted in lower metMb after eight days of storage. In this study, metMb formation was found to be lower than in the control group until the sixth day of storage (Figure 6). Oxidation inhibition was observed in the extract-added meat samples. The lower result compared to the literature can be explained by the larger surface area of minced meat compared to meatballs. This situation had an effect on the metMb formation of minced meat with natural antioxidant added.

Comparing the metMb and pH values, the efficiency of extract on quality was found to be significant (Figure 6). pH is effective on color, aroma and water retaining capacity. Consumers frequently associate bright-red color with beef freshness and wholesomeness. Higher than normal pH conditions are an example of a color deviation in which beef failed to have a bright-red color, leading to discounted carcasses and economic losses to the meat industry [37]. The change in pH of extract-marinated meat positively affected the quality characteristics. On the other hand, in studies on experimental model systems, the chlorogenic acid was reported to be among the effective polyphenols playing a role in metMb stability [9]. In the present study, a high rate of chlorogenic acid was found in the extract (11.18%).

Table 4 shows that effect from different references of artichoke (*C. scolymus*) powder on the quality performances on meat and meat products. Studies have had positive effects on quality at different levels in all meat products with added artichoke compared to our study. In our study, storage times of unprocessed meat products are less resistant to spoilage. When all the findings are interpreted together with the results of this study, phytochemicals can be used as a natural antioxidant agent of control during the storage period. Color plays an important role in both the quality and consumer acceptance of meat and meat products [38]. The instrumental color values were given in Table 5. The L* value was found to be slightly higher in added *C. scolymus* treatments than control samples (day zero and day three). Previous studies showed that the addition of various plant-based antioxidants did not change the L* values of meat and meat products [23]. The most important standard for the assessment of the oxidation is the “a*” value (redness), and decreased redness in meat is imagined as an index of oxidation. The a* values (redness) of minced meat ranges between 10.13 and 15.12. During the storage from the initial to the tenth day, redness of control and control samples decreased significantly (*p* < 0.05), but extract added treatments were higher than control samples and kept their redness. In a study in which plant-based antioxidants were used, including *C. scolymus* extract, decreased a* values were found, similar to our study findings. The b* values (yellowness) of minced meat ranged between 12.11 and 15.92. Significant reduction in the yellowness (b*) values were recorded in minced meat due to the added extract during storage. Different studies reported that the natural phenolic extracts showed no significant difference in L* and b* values but significant decrease in a* values during storage [23]. When the findings were compared with the literature, the color properties of the *C. scolymus* extract were found to be similar to the literature since they contained different plant-based extracts of the aforementioned compounds.

## 4. Conclusions

Results of the present study demonstrate using LC-QTOF-MS that artichoke (*C. scolymus*) powder extract contains high levels of quercetin and chlorogenic acid. The artichoke, which was found to have antioxidant properties, positively affected the chemical characteristics of the minced meat. It can be concluded that the added extract (as natural antioxidants) could successfully preserve antioxidant activity in minced meat stored at −18 °C for up to 10 days. *C. scolymus* powder with highly bioactive compounds improved the quality properties of minced meat samples during storage. It exhibited antimicrobial activity against pathogens that spoil food and are important for meat technology. MetMb levels could be maintained during storage. Hence, incorporation of extract stabilized the color of minced meat and had a significant impact on sensory characteristics during storage period. As a result, the *C. scolymus* powder extract can replace the chemical compounds in meat and meat products formulations to control the oxidative change and undesirable microbial activity. It can be applied as natural antioxidants to extend the shelf-life of meat products to achieve and valuable healthy meat products. In addition, the sensory and other quality parameters examined in more detailed research needs to be addressed in further studies.

## Figures and Tables

**Table 1:** Total phenolic and total flavonoid compounds.

Method of Extract.	Total Phenolic Compounds (mg GAE/g extract)	Total Flavonoid Compounds (mg QE/g extract)
Method 1	17.37 ± 0.07 ^aB^	6.45 ± 0.03 ^bB^
Method 2	98.26 ± 0.05 ^aA^	19.74 ± 0.04 ^bA^

^a,b^: Means followed by different letters within the same line represent significant differences (*p* < 0.05). ^A,B^: Means followed by different letters within the same column represent significant differences. Data are the average of triplicates.

**Table 2:** Method calibration and validation parameters for analysis of the phenolic standards.

Peak No	Phenolic Compounds	R_t_ (min)	Observed m/z	Recovery (%)	Rec.RSD (%)	R^2^	LOD (µg/mL)	LOQ (µg/mL)
**1**	3,4,5-Trihydroxybenzoic acid	0.475	400.1022	90.28 ± 2.24	1.76	0.9967	0.07	0.18
**2**	*trans*-3,4-Dihydroxycinnamic acid	0.850	455.1171	88.27 ± 4.37	3.42	0.9996	0.11	0.35
**3**	4-Hydroxy-3-methoxybenzoic acid	1.18	388.2636	90.51 ± 2.10	1.63	0.9985	0.06	0.13
**4**	3,5-Dimethoxy-4-hydroxybenzoic acid	1.75	392.2331	90.38 ± 2.16	1.69	0.9967	0.06	0.16
**5**	*trans*-4-Hydroxycinnamic acid	1.9	371.1338	90.53 ± 2.03	1.58	0.9988	0.06	0.13
**6**	p-Coumaroyl-*O*-feruloylquinic acid I	2.02	266.2013	90.99 ± 0.36	0.27	0.9978	0.005	0.01
**7**	1,4,5-Trihydroxycyclohexanecarboxylic acid 3-(3,4-dihydroxycinnamate)	2.75	481.1711	85.18 ± 6.27	4.92	0.9994	0.15	0.51
**8**	(+)-Catechin	2.83	461.1035	87.32 ± 4.90	3.85	0.9991	0.12	0.42
**9**	(−)-Epigallocatechin	2.93	423.1521	89.39 ± 3.33	2.61	0.9967	0.08	0.27
**10**	p-Coumaroyl-*O*-feruloylquinic acid II	3.04	362.2082	90.70 ± 1.49	1.17	0.9980	0.05	0.11
**11**	*trans*-4-Hydroxy-3-methoxycinnamic acid	3.62	298.1291	90.83 ± 1.30	1.00	0.9985	0.03	0.07
**12**	*trans*-4-Hydroxy-3-methoxycinnamic acid	4.33	466.1588	85.96 ± 5.88	4.61	0.9964	0.13	0.48
**13**	p-Coumaroyl-*O*-feruloylquinic acid III	4.52	403.1022	90.04 ± 2.67	2.09	0.9985	0.07	0.20
**14**	4′,5,7-Trihydroxyflavone	5.36	321.3371	90.77 ± 1.41	1.09	0.9980	0.04	0.09
**15**	p-Coumaroyl-*O*-feruloylquinic acid IV	5.92	286.3122	90.90 ± 0.88	0.68	0.9966	0.01	0.06
**16**	7-neohesperidoside	6.65	448.1822	88.53 ± 3.94	3.11	0.9973	0.10	0.35
**17**	2-(3,4-Dihydroxyphenyl)-3,5,7-trihydroxy-4H-1-benzopyran-4-one	7.97	488.1088	84.95 ± 6.52	5.11	0.9973	0.15	0.52
**18**	3,4′,5-Trihydroxy-*trans*-stilbene	8.5	411.2633	89.74 ± 3.20	2.49	0.9988	0.08	0.22
**19**	p-Coumaroyl-*O*-feruloylquinic acid V	8.84	273.1023	90.95 ± 0.63	0.49	0.9985	0.01	0.04

LOD: limit of detection; LOQ: limit of quantification; Rec.: recovery; RSD: relative standard deviation.

**Table 3:** Antimicrobial activity of the added *C. scolymus* powder on meat extract solution.

Treatments	Inhibition Zone (mm)				
	*E. coli*	*S. aureus*	*L. monocytogenes*	*S. typhimurium*	*E. faecalis*
Added *+ C. scolymus* (5%)	19.74 ± 0.08 ^cB^	19.75 ± 1.09 ^cC^	17.32 ± 0.83 ^dB^	29.33 ± 1.13 ^aB^	25.20 ± 0.95 ^bB^
Added + *C. scolymus* (10%)	20.07 ± 0.66 ^cB^	21.10 ± 0.92 ^cB^	18.05 ± 0.45 ^dB^	30.95 ± 0.88 ^aB^	25.37 ± 0.53 ^bB^
Control(+)	29.00 ± 1.41 ^bA^	30.00 ± 0.46 ^bA^	27.00 ± 0.95 ^cA^	32.00 ± 0.62 ^aA^	31.50 ± 1.15 ^aA^
Control(−)	0	0	0	0	0

Values are expressed as means ± SD. Standard disk; 6mm, control(+); ampicillin, control(−); distilled water, ^a,b,c,d^: Means followed by different letters within the same line represent significant differences (*p* < 0.05). ^A,B,C^: Means followed by different letters within the same column represent significant differences. Data are the average of triplicates.

**Table 4:** Effect from different references of artichoke (*C. scolymus)* powder on the quality performances on meat products.

Added-Extract	Foodstuffs	Storage Period	Quality Measurements	Results	Reference
Artichoke (*C. scolymus)* powder extract	Minced Meat on the During Frozen Storage	10 days	Antioxidant Activity (DPPH, FRAP, ABTS) Antimicrobial Activity metMb Reducing Activity	A potential to improve the meat quality	In this study
Artichoke (*C. scolymus)* leaf powder	Meat Quality of Japanese Quail	21 days	TBARS WHC	A potential to improve the oxidative stability and meat quality	[39]
Artichoke (*C. scolymus)* leaf powder	Frozen Meat Quality of Japanese Quail	21 days	TBARS WHC	A potential to improve the oxidative stability and meat quality	[40]
Artichoke (*C. scolymus)* extract	Chicken thigh meat	35 days	Antioxidant activity (DPPH)	Decreases GPx and CAT activities in Chicken meat	[33]
Artichoke (*C. scolymus)* byproducts extracts	Raw beef patties during refrigerated storage	7 days	Antioxidant activity (DPPH) TBARS	As natural antioxidant in meat products	[23]

TBARS: 2-thiobarbituric acid-reactive substance, WHC: Water holding capacity, GPx: glutathione peroxidase, CAT: catalase.

**Table 5:** Color parameters (L*, a*, b*) of minced meat samples during storage.

Storage Period	Storage Period	L*	a*	b*
Control	Day 0	35.62 ± 0.32 ^aB^	14.67 ± 0.48 ^bA^	15.03 ± 0.57 ^bA^
Added *+ C. scolymus*		36.84 ± 0.51 ^aA^	15.12 ± 0.37 ^bA^	15.92 ± 0.63 ^bA^
Control	Day 3	35.74 ± 0.43 ^aB^	12.28 ± 0.51 ^bA^	14.22 ± 0.39 ^bA^
Added *+ C. scolymus*		36.21 ± 0.31 ^aA^	12.88 ± 0.31 ^bA^	14.20 ± 0.46 ^bA^
Control	Day 6	37.13 ± 0.46 ^aA^	10.67 ± 0.54 ^bB^	13.44 ± 0.63 ^bB^
Added *+ C. scolymus*		35.24 ± 0.36 ^aB^	10.75 ± 0.49 ^bB^	12.88 ± 0.38 ^bB^
Control	Day 10	38.56 ± 0.34 ^aA^	9.93 ± 0.48 ^bB^	12.82 ± 0.51 ^bB^
Added *+ C. scolymus*		34.88 ± 0.38 ^aB^	10.13 ± 0.56 ^bB^	12.11 ± 0.34 ^bB^

^a,b^: Means followed by different letters within the same line represent significant differences (*p* < 0.05). ^A,B^: Means followed by different letters within the same column represent significant differences. Data are the average of triplicates.

## Data Availability

The data are available by the corresponding author upon.

## Sample Availability

Samples of the compounds are available from the authors.
